# Crystallographic evidence for unintended benziso­thia­zolinone 1-oxide formation from benzo­thia­zinones through oxidation

**DOI:** 10.1107/S2053229620010931

**Published:** 2020-08-21

**Authors:** Tamira Eckhardt, Richard Goddard, Christoph Lehmann, Adrian Richter, Henok Asfaw Sahile, Rui Liu, Rohit Tiwari, Allen G. Oliver, Marvin J. Miller, Rüdiger W. Seidel, Peter Imming

**Affiliations:** a Institut für Pharmazie, Martin-Luther-Universität Halle-Wittenberg, Wolfgang-Langenbeck-Strasse 4, 06120 Halle (Saale), Germany; b Max-Planck-Institut für Kohlenforschung, Kaiser-Wilhelm-Platz 1, 45470 Mülheim an der Ruhr, Germany; c Department of Medicine and Department of Microbiology and Immunology, University of British Columbia, Vancouver, British Columbia, V6T 1Z3, Canada; d Department of Chemistry and Biochemistry, University of Notre Dame, Indiana 46556, USA

**Keywords:** benzo­thia­zinone, BTZ043, benziso­thia­zolinone, ring contraction, crystal structure, anti­mycobacterial activity

## Abstract

1,3-Benzo­thia­zin-4-ones (BTZs) are a promising new class of drugs with activity against *Mycobacterium tuberculosis*, which have already reached clinical trials. A product obtained in low yield upon treatment of 8-nitro-2-(piperidin-1-yl)-6-(tri­fluoro­meth­yl)-4*H*-benzo­thia­zin-4-one with 3-chloro­perbenzoic acid, in ana­logy to a literature report describing the formation of sulfoxide and sulfone derived from BTZ043 [Tiwari *et al.* (2015 ▸). *ACS Med. Chem. Lett.* **6**, 128–133], is a ring-contracted benziso­thia­zolinone (BIT) 1-oxide, namely, 7-nitro-2-(piperi­dine-1-carbon­yl)-5-(tri­fluoro­meth­yl)benzo[*d*]iso­thia­zol-3(2*H*)-one 1-oxide, C_14_H_12_F_3_N_3_O_5_S, as revealed by X-ray crystallography. Single-crystal X-ray analysis of the oxidation product originally assigned as BTZ043 sulfone provides clear evidence that the structure of the purported BTZ043 sulfone is likewise the corresponding BIT 1-oxide, namely, 2-[(*S*)-2-methyl-1,4-dioxa-8-aza­spiro­[4.5]decane-8-carbon­yl]-7-nitro-5-(tri­fluoro­meth­yl)benzo[*d*]iso­thia­zol-3(2*H*)-one 1-oxide, C_17_H_16_F_3_N_3_O_7_S. A possible mechanism for the ring contraction affording the BIT 1-oxides instead of the anti­cipated constitutionally isomeric BTZ sulfones and anti­mycobacterial activities thereof are discussed.

## Abstract 2

X-ray crystallography revealed the unintended formation of benziso­thia­zolinone 1-oxides from 1,3-benzo­thia­zin-4-ones through oxidation instead of the anti­cipated benzo­thia­zinone sulfones, which would be constitutional isomers.

## Introduction   

Due to extremely low cidal concentrations against *Mycobacterium tuberculosis in vitro*, 8-nitro-1,3-benzo­thia­zin-4-ones (BTZs) have been the focus of many chemical, pharmacological and, recently, clinical studies (Mikušová *et al.*, 2014 ▸; Kloss *et al.*, 2017 ▸; Makarov & Mikušová, 2020 ▸). Several promising com­pounds with improved aqueous solubilities have been identified with potent anti­tubercular activity (Zhang *et al.*, 2019 ▸). The first small mol­ecule crystal structure of a BTZ, namely macozinone (PBTZ169), was reported in this journal by Zhang & Aldrich (2019 ▸). So far, the excellent *in vitro* activity appears not to translate to the low daily doses aspired for a medication that needs to be administered for months (Lupien *et al.*, 2018 ▸). This could be attributed to pharmacokinetic problems and rapid metabolism by gut bacteria (Lv *et al.*, 2017 ▸).

Research inter­est in this com­pound class is also inspired by the chemical versatility of the BTZs, which offer several points of attack, especially for nucleophiles and reducing agents (Tiwari *et al.*, 2013 ▸). In turn, the BTZ S atom appears to be not very susceptible to oxidation. When BTZ043 (Scheme 1) was treated with the oxidizing agent 3-chloro­perbenzoic acid at room temperature for several days, a major amount of unreacted BTZ starting material was recovered and small qu­anti­ties of two oxidation products were isolated. Based on ^1^H NMR spectroscopy and the sum formula calculated from high-resolution mass spectrometry, the corresponding BTZ sulfoxide and sulfone structures were assigned (Tiwari *et al.*, 2015 ▸).
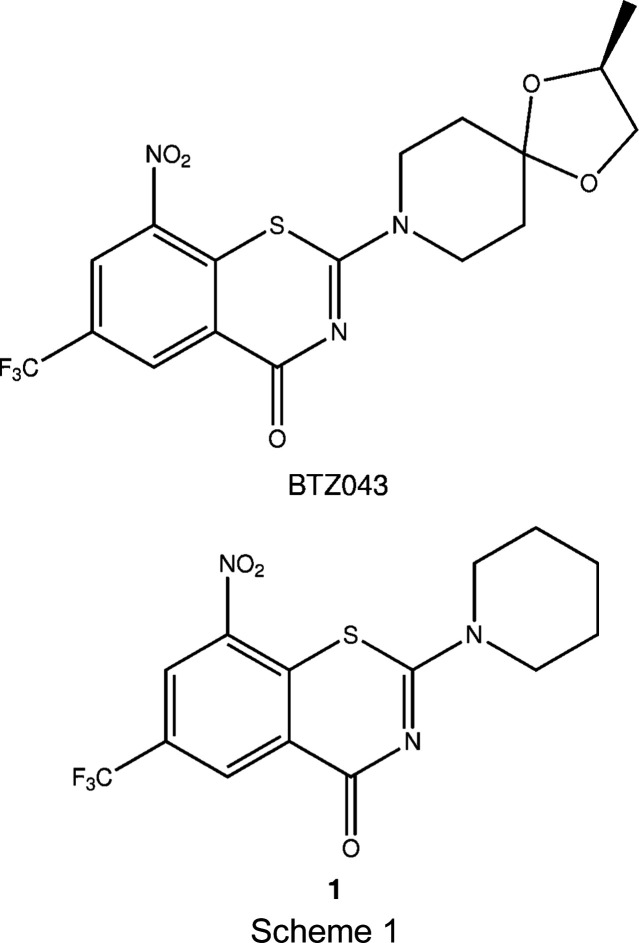


**Figure 1:**
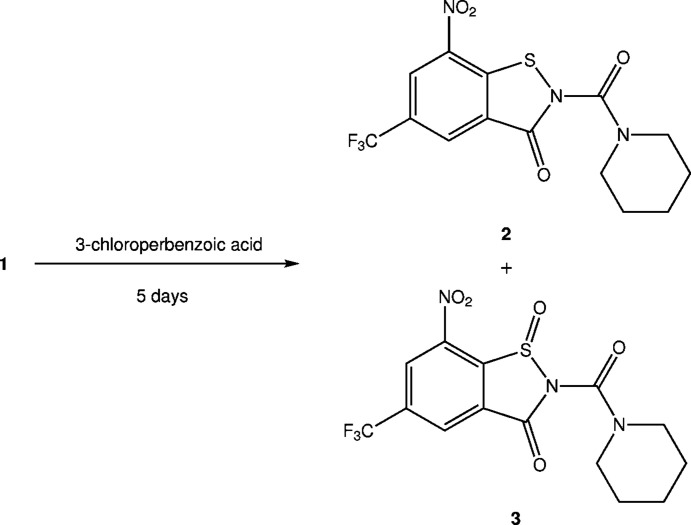
Ring-contracted oxidation products resulting from the treatment of **1** with 3-chloro­perbenzoic acid at room temperature.

Treatment of 8-nitro-2-(piperidin-1-yl)-6-(tri­fluoro­meth­yl)-4*H*-benzo­thia­zin-4-one (**1**, Scheme 1) with 3-chloro­perbenzoic acid in a similar way and crystallographic characterization of one of the oxidation products revealed the formation of a ring-contracted benziso­thia­zolone (BIT) 1-oxide instead of the anti­ci­pated BTZ sulfone (Fig. 1 ▸). Subsequent crystallographic reinvestigation of the BTZ043 oxidation product originally described as BTZ sulfone by us (Tiwari *et al.*, 2015 ▸) evidenced that the structure must be revised to the corresponding ring-contracted BIT 1-oxide. In this article, we report the structural characterization of BIT 1-oxides resulting from oxidation of **1** and BTZ043, and propose a reaction mechanism of the ring contraction. We furthermore show by analysis of spectroscopic data and deliberate synthesis that the purported BTZ sulfoxide is actually a BIT.

## Experimental   

### General   

Starting materials were obtained from commercial sources and were used as received. Solvents were of analytical grade. Compound **1** was synthesized as described elsewhere (Rudolph *et al.*, 2016 ▸). Thin-layer chromatography (TLC) was performed on Silica gel 60 F_254_ TLC plates (Merck KGaA, Darmstadt). The reported *R*_F_ values are uncorrected. Flash chromatography was carried out with a 40 g puriFlash column (30 µm silica gel, 60 Å, 500 m^2^ g^−1^, Inter­chim, Montluçon, France). Preparative HPLC was performed on a Shimadzu LC-10AD system using 19 × 150 mm XTerra RP-18 columns (7 µm, Waters, Milford, Massachusetts, USA). ^1^H and ^13^C NMR spectra were recorded at room temperature on an Agilent Technologies VNMRS 400 MHz NMR spectrometer (*bs* = broad singlet, *q* = quartet and *m* = multiplet). Chemical shifts are referenced to the residual signals of CDCl_3_ (δ_H_ = 7.26 ppm and δ_C_ = 77.0 ppm). High-resolution mass spectra (HRMS) were measured on a Bruker Daltonics APEXIII FT–ICR mass spectrometer.

### Synthesis and crystallization   

Compounds **2** and **3** were obtained when **1** was treated with 3-chloro­perbenzoic acid, adapting the procedure described by Tiwari *et al.* (2015 ▸). A solution of 3-chloro­perbenzoic acid (1.04 g, 6.0 mmol) in di­chloro­methane (6.5 ml) was added drop­wise to a stirred solution of 8-nitro-2-(piperidin-1-yl)-6-(tri­fluoromethyl)-4*H*-1,3-benzo­thia­zin-4-one, **1** (1.09 g, 3.0 mmol), in di­chloro­methane (5 ml) at 0 °C. After stirring for 4 d at room temperature, additional 3-chloro­perbenzoic acid (0.5 g) was added and the mixture was stirred for another day. The resulting mixture was washed twice with a saturated sodium bicarbonate solution (55 ml) and then once with deionized water (55 ml). After drying over sodium sulfate, the solvent was removed using a rotary evaporator. The crude product was subjected to flash chromatography [gradient of 50–100 (*v*/*v*) ethyl acetate/hepta­ne] to give **2** and **3**. Both com­pounds were purified by HPLC [gradient of 5–95 (*v*/*v*) aceto­nitrile/water in 10 min + 0.05% tri­fluoro­acetic acid] to yield 6 mg of **2** (0.016 mmol, 0.5%) and 35 mg of **3** (0.089 mmol, 3%).

Crystals of **3** suitable for single-crystal X-ray analysis were obtained after a couple of days when a solution of *ca* 5 mg of the com­pound in ethanol (1.5 ml) in a 10 × 50 mm glass vial with a screw cap was left at room temperature and the solvent allowed to evaporate slowly.

The synthesis of **4** has been reported elsewhere (Tiwari *et al.*, 2015 ▸; therein mistaken for the sulfone of the BTZ043 starting material). For the preparation of crystals suitable for single-crystal X-ray analysis, the com­pound (1 mg) was added to a 6 × 50 mm round-bottomed borosilicate glass culture tube, and dissolved in chloro­form (0.4 ml) to give a clear homogenous solution. The tube was placed in a 20 ml scintillation vial, followed by the addition of pentane (5 ml). The vial was capped tightly and the resulting diffusion chamber was allowed to stand undisturbed at room temperature. After several days, crystals suitable for X-ray analysis formed.

#### Analytical data for 2   

^1^H NMR (400 MHz, CDCl_3_): δ 8.77 (*bs*, 1H), 8.57 (*bs*, 1H), 3.58 (*m*, 4H), 1.78–1.70 (*m*, 6H) ppm; HRMS(ESI): calculated for C_14_H_12_F_3_N_3_O_4_S [*M* + Na]^+^ 398.0398, found 398.0397; *R*_F_ = 0.29 (ethyl acetate/heptane, 2:8 *v*/*v*).

#### Analytical data for 3   

^1^H NMR (400 MHz, CDCl_3_) δ 8.79 (*bs*, 1H), 8.58 (*bs*, 1H), 3.68–3.51 (*m*, 4H), 1.81–1.62 (*m*, 6H) ppm; ^13^C NMR (101 MHz, CDCl_3_): δ 159.5, 148.3, 144.8, 143.0, 137.8 (*q*, ^2^ *J*_C,F_ = 35.5 Hz), 132.8, 129.4 (*q*, ^3^ *J*_C,F_ = 3.6 Hz), 126.6 (*q*, ^3^ *J*_C,F_ = 3.6 Hz), 121.6 (*q*, ^1^ *J*_C,F_ = 274.2 Hz), 47.5, 25.8, 24.0 ppm; HRMS(ESI): calculated for C_14_H_12_F_3_N_3_O_5_S [*M* + H]^+^ 392.0528, found 392.0526; *R*_F_ = 0.22 (ethyl acetate/heptane, 2:8 *v*/*v*).

### Refinement   

Crystal data, data collection and structure refinement details are summarized in Table 1 ▸. H-atom positions were calculated geometrically, with aromatic C—H = 0.95 Å, methyl C—H = 0.98 Å, methyl­ene C—H = 0.99 Å and methine C—H = 1.00 Å, and refined using a riding model, with *U*_iso_(H) = 1.2*U*_eq_(C) (1.5 for methyl groups). The torsion angles of the methyl groups were initially determined using a circular Fourier search and subsequently refined while maintaining the tetra­hedral structure.

## Results and discussion   

### Synthesis and structural identification   

When **1** was treated with 3-chloro­perbenzoic acid, adapting the procedure for BTZ043 reported by Tiwari *et al.* (2015 ▸), likewise a major amount of the BTZ starting material was recovered, but small qu­anti­ties of oxidation products **2** and **3** could be isolated by chromatography. The corresponding sum formulae were obtained from high-resolution mass spectra, and **3** was subjected to X-ray crystallography. The X-ray analysis umambiguously revealed the BIT 1-oxide structure for **3** instead of the anti­cipated BTZ sulfone, which would be a constitutional isomer (Fig. 1 ▸). Accordingly, and in agreement with the sum formula, we propose the corresponding BIT structure for **2** instead of the anti­cipated BTZ sulfoxide, which likewise would be a constitutional isomer.

**Figure 2:**
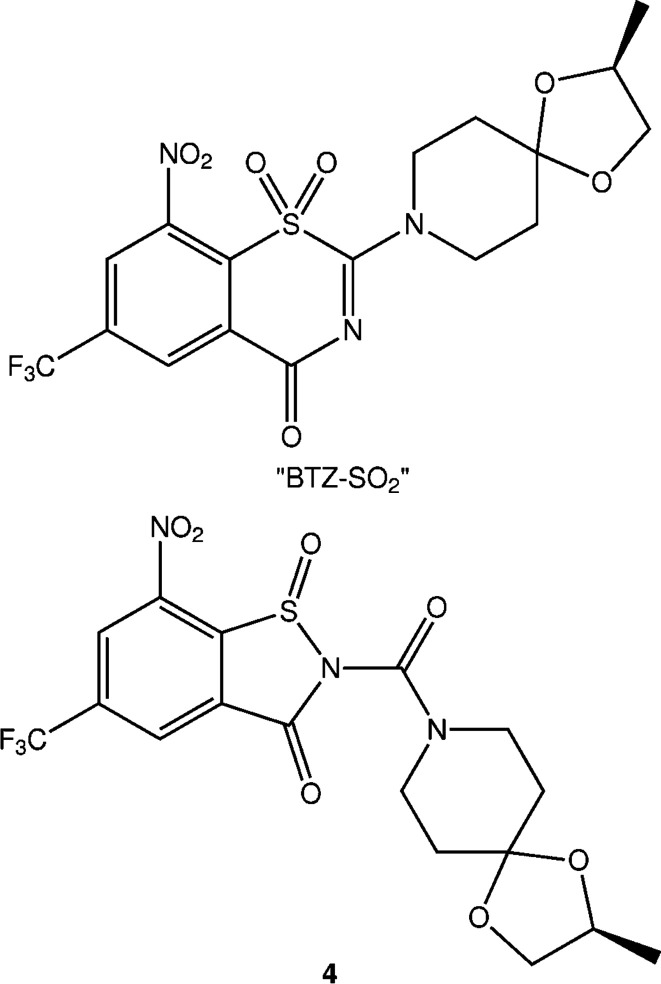
The incorrect structure ‘BTZ-SO_2_’ (Tiwari *et al.*, 2015 ▸) and the revised structure of **4**, resulting from treatment of BTZ043 with 3-chloro­perbenzoic acid at room temperature.

Single-crystal X-ray analysis of the oxidation product of BTZ043 resulting from treatment with 3-chloro­perbenzoic acid, which was named ‘BTZ-SO_2_’ by Tiwari *et al.* (2015 ▸), provided clear evidence for the formation of the corresponding BIT 1-oxide **4**, a constitutional isomer of the reported BTZ sulfone (Fig. 2 ▸).

Table 2 ▸ com­pares the ^1^H NMR shifts of the two aromatic protons in **1** and BTZ043 with those of the derived oxidation products. For both **2** and **3**, as well as ‘BTZ-SO’ and **4**, the signals assigned to the two aromatic protons are upfield shifted com­pared with the parent BTZs. While assuming the anti­cipated BTZ sulfoxide and sulfone structures, Tiwari *et al.* (2015 ▸) attributed this effect to the influence of the S-atom lone-pair delocalization and the loss of aromaticity due to the nonplanarity of the 1,4-thia­zinone rings in the assumed BTZ sulfoxide and sulfone structures. Higher electron density within the encountered BIT nine-membered heterobicyclic system, as com­pared with the BTZ ten-membered system, however, provides a better explanation for the shielding of the aromatic protons resulting in the observed upfield shifts. For further corroboration, BIT **2**, for which we did not obtain crystals suitable for single-crystal X-ray analysis, was synthesized deliberately from 2-chloro-3-nitro-5-(tri­fluoro­meth­yl)nitro­benzamide (see supporting information), following an established procedure for related BITs (Bhakuni *et al.*, 2012 ▸). NMR spectroscopic and mass spectrometric data of the product thus obtained agreed with those for **2** resulting from treatment of **1** with 3-chloro­perbenzoic acid.

### Structural descriptions of 3 and 4   

**Figure 3:**
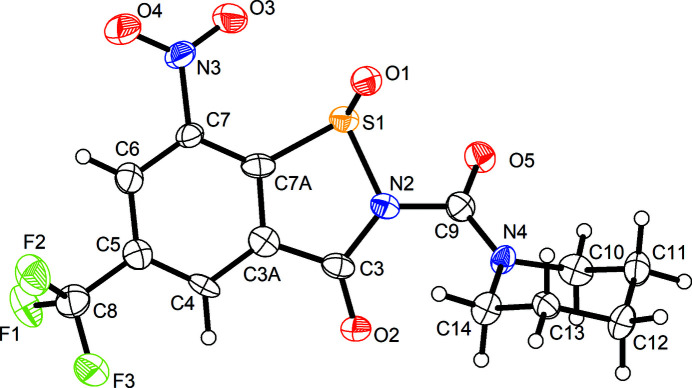
The mol­ecular structure of **3** in the crystal. Displacement ellipsoids are drawn at the 50% probability level. H atoms are represented by small spheres of arbitrary radii.

Compound **3** crystallizes in the polar ortho­rhom­bic space group *Iba*2 with one mol­ecule in the asymmetric unit (*Z*′ = 1). Fig. 3 ▸ shows the mol­ecular structure in the crystal. The BIT system is not entirely planar. Atoms N2 and O2 are displaced by 0.27 (1) and −0.20 (1) Å, respectively, above and below the mean plane defined by the benzene ring. The plane of the nitro group is tilted out of this plane by 12 (1)°. The sulfinamide moiety exhibits a pyramidal structure at the S atom, as expected. The mol­ecule in the chosen asymmetric unit is *R*-configured at the S atom. It is worth emphasizing, however, that the *S* enanti­omer is generated by glide symmetry in the polar crystal structure so the crystal is a racemate. The central carbamide moiety is tilted out of the BIT plane, as revealed by the torsion angles about the N2—C9 bond. The structure at atom N2 is slightly pyramidal, whereas that at N4 is virtually planar due to conjugation with the adjacent carbonyl group. The piperidine ring adopts a low-energy chair conformation with some deviations of the bond angles from ideal tetra­hedal angles, which can be attributed to the planarity at N4.

**Figure 4:**
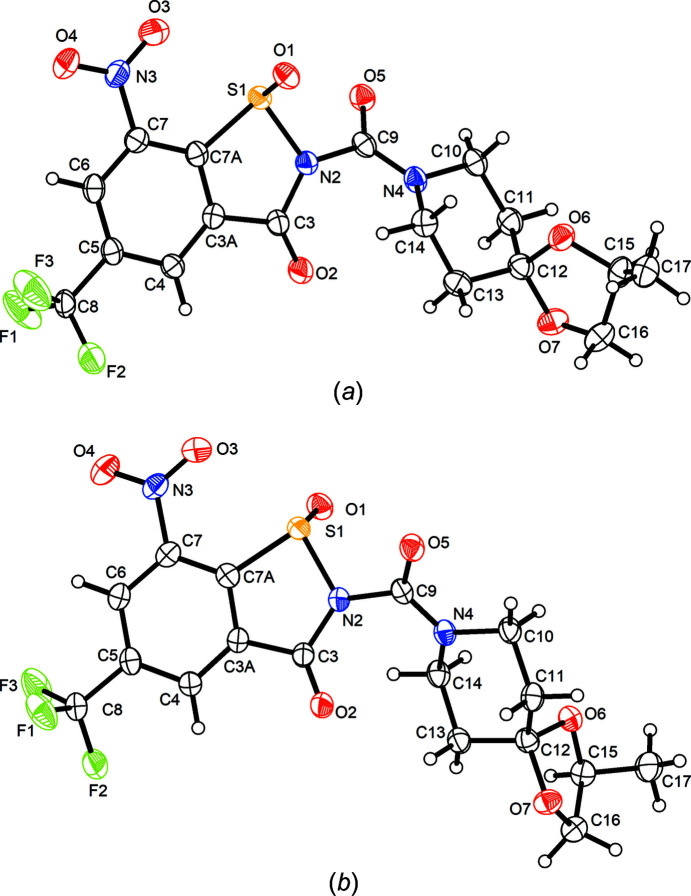
Displacement ellipsoid plots (50% probability level) of the two crystallographically distinct diastereomeric mol­ecules of **4**. H atoms are represented by small spheres of arbitrary radii.

Compound **4** crystallizes in the Sohncke space group *P*2_1_ with two diastereomers in the asymmetric unit (*Z*′ = 2). Fig. 4 ▸ depicts displacement ellipsoid plots for both unique mol­ecules. Compared with **3**, com­pound **4** exhibits an additional spiro-(2*S*)-methyl-1,3-dioxolane group appended to the piperidine ring in the 4-position. The *S* configuration at C15, as in the BTZ043 starting material, is encountered in both crystallographically distinct mol­ecules and the configurational assignment was confirmed by a Flack *x* parameter (Parsons *et al.*, 2013 ▸) close to zero with a reasonably small standard uncertainty (Table 1 ▸). The two independent mol­ecules exhibit opposite configurations at the S atoms and thus the crystal is a cocrystal of two diastereomers. Possible causes of *Z*′ > 1 crystallization have been discussed (Steed & Steed, 2015 ▸). Here, *Z*′ = 2 is attributed to diasteromeric crystallization. The formation of a diastereomeric conglomerate or a solid solution would have been an alternative crystallization pathway. Apart from the configuration at the S atom, the distinct mol­ecules also exhibit different conformations of the 1,3-dioxolane five-membered rings. In mol­ecule 1 (Fig. 4 ▸ *a*), the 1,3-dioxolane ring adopts an envelope conformation with atom C16 on the flap, whereas in mol­ecule 2 (Fig. 4 ▸ *b*), the ring is close to an envelope with the spiro atom C12 on the flap. As in **3**, the BIT systems deviate slightly from planarity. In mol­ecule 1, atom O2 is displaced from the mean plane of the benzene ring by 0.323 (6) Å, and in mol­ecule 2, atoms N2 and O2 deviate by −0.118 (5) and 0.333 (5) Å, respectively, from this plane. The tilt angle between the mean plane of the benzene ring and the plane of the nitro group is 16.4 (3)° in mol­ecule 1 and 10.7 (4)° in mol­ecule 2. Similar to **3**, in both mol­ecules, the central carbamide moiety is tilted out of the plane of the BIT skeleton and the appended piperidine ring adopts a low-energy chair conformation with some minor deviations of the bond angles.

**Figure 5:**
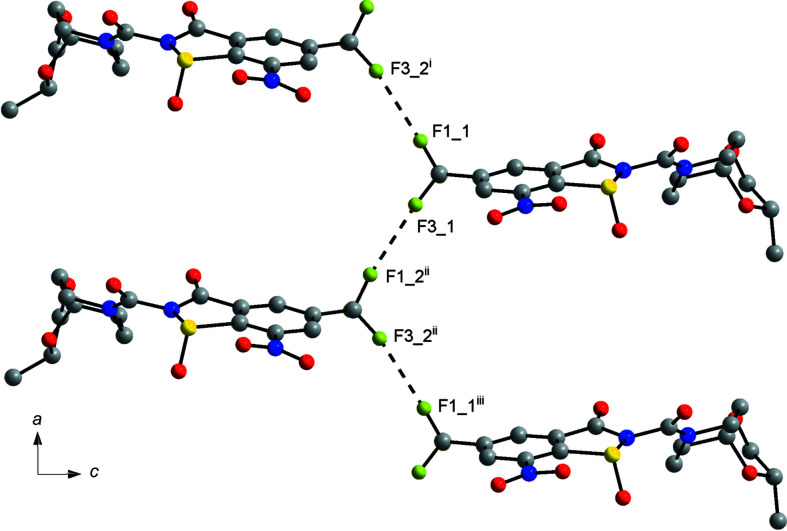
Part of the crystal structure of **4**, showing C—F⋯F—C contacts (dashed lines), viewed down the *b*-axis direction towards the origin. [Symmetry codes: (i) *x*, *y*, *z* − 1; (ii) *x* − 1, *y*, *z* − 1; (iii) *x* − 1, *y*, *z*.]

The supra­molecular structure of **4** in the solid state features short C—F⋯F—C contacts [F1_1⋯F3_2^i^ = 2.737 (4) Å and F3_1⋯F1_2^ii^ = 2.751 (4) Å)], which link unique mol­ecules 1 and 2 along the [100] direction (Fig. 5 ▸). According to the corresponding C—F⋯F angles in the range of 157.8–167.8°, these contacts may be classified as type-I F⋯ F inter­actions (Baker *et al.*, 2012 ▸). F⋯F contacts that are shorter than the sum of the van der Waals radii are not encountered in the crystal structure of **3**, but instead several short C—H⋯F contacts are observed (not depicted).

### Mechanistic discussion of the ring contraction   

**Figure 6:**
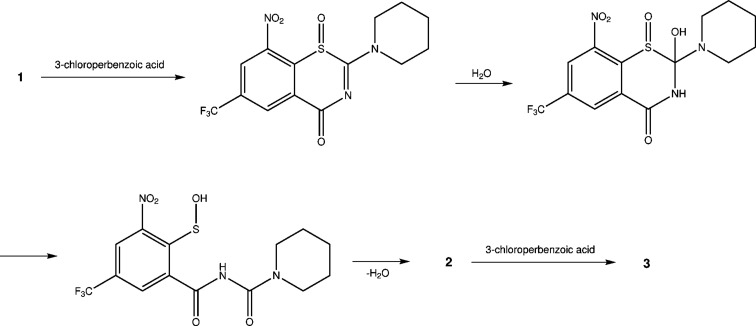
Postulated reaction mechanism for the formation of BITs and BIT 1-oxides from BTZs upon treatment with 3-chloro­perbenzoic acid (shown for **1**).

Since the ring-contracted oxidation products only formed in very low yields, investigation of the reaction mechanism of BTZ oxidation and rearrangement upon treatment with 3-chloro­perbenzoic acid was not undertaken. We propose the sequence shown in Fig. 6 ▸. This is in part based on a mechanism postulated by Szabó *et al.* (1988 ▸). We follow these authors in assuming that the anti­cipated oxidation of **1** to the corresponding BTZ sulfoxide occurred initially and was followed by nucleophilic addition of water (from wet 3-chloro­perbenzoic acid used) to the C=N bond of the BTZ system. Ring opening and rearrangement to a sulfenic acid group and an *N*-acyl­carbamide moiety within the mol­ecule would be followed by the loss of water to form **2**, which was then oxidized by another equivalent of 3-chloro­perbenzoic acid leading to **3**, which we isolated and structurally characterized by X-ray crystallog­raphy. Although this mechanism is only postulated, it explains why both **2** and **3** were formed.

### Anti­mycobacterial activities   

Tiwari *et al.* (2015 ▸) reported *in vitro* activities of the oxidation products against *Mycobacterium tuberculosis* and *M. aurum*, among other mycobacteria, albeit assuming the BTZ sulfoxide and sulfone structures, which are revised in the present work. We also evaluated the activities of **2** and **3** against *M. tuberculosis* and *M. aurum* (the assay protocols can be found in the supporting information). Although the structures of **2** and **3** differ from those of ‘BTZ-SO’ and **4** by the absence of the spiro-(2*S*)-methyl-1,3-dioxolane group appended to the piperi­dine ring in the 4-position, their activities against *M. tuberculosis* and *M. aurum* are com­parable (Table 3 ▸). Indeed, BITs are known to have anti­microbial activity and are used as preservatives (Novick *et al.*, 2013 ▸). Inter­estingly, BIT **2** and its 1-oxide **3**, as well as ‘BTZ-SO’ and **4**, show com­parable or better activity against both mycobacterial species than the corresponding BTZs **1** and BTZ043 (Table 3 ▸). Thus, BITs could likewise be considered as decaprenylphosphoryl-β-d-ribose 2′-epimerase (DprE1) in­hibi­tors, and work along this line is in progess. It should be noted, however, that BITs are known to have various mol­ecular targets in microorganisms (Gopinath *et al.*, 2017 ▸). This may render them less promising for the development of anti­mycobacterial agents.

## Supplementary Material

Crystal structure: contains datablock(s) global, 3, 4. DOI: 10.1107/S2053229620010931/ep3008sup1.cif

Structure factors: contains datablock(s) 3. DOI: 10.1107/S2053229620010931/ep30083sup2.hkl

Structure factors: contains datablock(s) 4. DOI: 10.1107/S2053229620010931/ep30084sup3.hkl

Supporting information file. DOI: 10.1107/S2053229620010931/ep30083sup4.cml

Supporting information file. DOI: 10.1107/S2053229620010931/ep30084sup5.cml

Description of both the deliberate synthesis of 2 and the antimycobacterial assays. DOI: 10.1107/S2053229620010931/ep3008sup6.pdf

CCDC references: 2022514, 2022515

## Figures and Tables

**Table 1:** Experimental details Experiments were carried out with Cu *K*α radiation. Refinement in both cases was with 1 restraint. H-atom parameters were constrained.

	**3**	**4**
Crystal data		
Chemical formula	C_14_H_12_F_3_N_3_O_5_S	C_17_H_16_F_3_N_3_O_7_S
*M*_r_	391.33	463.39
Crystal system, space group	Orthorhombic, *I* *b* *a*2	Monoclinic, *P*2_1_
Temperature (K)	100	120
*a*, *b*, *c* (Å)	17.6719 (8), 25.7296 (12), 6.8887 (3)	8.8165 (2), 16.1649 (4), 13.4246 (3)
α, β, γ (°)	90, 90, 90	90, 90.0022 (12), 90
*V* (Å^3^)	3132.2 (2)	1913.24 (8)
*Z*	8	4
μ (mm^−1^)	2.50	2.23
Crystal size (mm)	0.23 × 0.06 × 0.04	0.25 × 0.07 × 0.04

Data collection		
Diffractometer	Bruker Kappa Mach3 APEXII	Bruker PHOTON-II
Absorption correction	Gaussian (*SADABS*; Bruker, 2012 ▸)	Numerical (*SADABS*; Bruker, 2012 ▸)
*T*_min_, *T*_max_	0.748, 0.936	0.715, 0.949
No. of measured, independent and observed [*I* > 2σ(*I*)] reflections	22457, 2477, 1843	36857, 7140, 6860
*R*_int_	0.102	0.041
(sin θ/λ)_max_ (Å^−1^)	0.610	0.611

Refinement		
*R*[*F* ^2^ > 2σ(*F* ^2^)], *wR*(*F* ^2^), *S*	0.059, 0.170, 1.08	0.033, 0.083, 1.04
No. of reflections	2477	7140
No. of parameters	235	561
Δρ_max_, Δρ_min_ (e Å^−3^)	0.53, −0.75	0.61, −0.31
Absolute structure	Flack *x* determined using 550 quotients [(*I* ^+^) − (*I* ^−^)]/[(*I* ^+^) + (*I* ^−^)] (Parsons *et al.*, 2013 ▸)	Flack *x* determined using 3080 quotients [(*I* ^+^) − (*I* ^−^)]/[(*I* ^+^) + (*I* ^−^)] (Parsons *et al.*, 2013 ▸)
Absolute structure parameter	0.08 (5)	0.017 (6)

Computer programs: *APEX3* (Bruker, 2015 ▸), *SAINT* (Bruker, 2015 ▸), *SHELXT* (Sheldrick, 2015*a* ▸), *SHELXL2018* (Sheldrick, 2015*b* ▸), *DIAMOND* (Brandenburg, 2018 ▸), *enCIFer* (Allen *et al.*, 2004 ▸) and *publCIF* (Westrip, 2010 ▸).

**Table 2:** ^1^H NMR shifts (ppm) of the aromatic protons in CDCl_3_ for **1**–**3**, BTZ043 and its oxidation products Data for **1** were taken from Rudolph *et al.* (2016 ▸) and data for BTZ043, ‘BTZ-SO’ and ‘BTZ-SO_2_’ were taken from Tiwari *et al.* (2015 ▸).

**1**	**2**	**3**	BTZ043	‘BTZ-SO’	‘BTZ-SO_2_’ (**4**)
9.08	8.77	8.79	9.02	8.78	8.80
8.72	8.57	8.58	8.55	8.59	8.58

**Table 3:** *In vitro* activities (MIC_90_ in µ*M*) of **1**-**3**, BTZ043 and its oxidation products Data for BTZ043, ‘BTZ-SO’ and ‘BTZ-SO_2_’ were taken from Tiwari *et al.* (2015 ▸).

	**1**	**2**	**3**	BTZ043	‘BTZ-SO’	‘BTZ-SO_2_’ (**4**)
*M. tuberculosis*	4.3^*a*^	<0.26^*a*^	8.0^*a*^	0.02^*b*^	0.06^*b*^	0.48^*b*^
*M. aurum*	10.9^*c*^	2.0^*c*^	19.4^*c*^	>200^*a*^	3.13–12.5^*d*^	>200^*d*^

Notes: (*a*) *M. tuberculosis* H_37_Rv pTEC27 (7H9 medium plus 10% OADC and 0.05% polysorbate 80, microplate RFP assay); (*b*) *M. tuberculosis* H_37_Rv (7H9 medium plus casitone, palmitic acid, albumin and catalase; MABA, Microplate Alamar Blue Assay); (*c*) *M. aurum* DSMZ 43999 (7H9 medium plus 10% OADC and 0.5% glycerol, microplate OD600 Assay); (*d*) *M. aurum* SB66.
