# Supplementary material for: Crystallographic evidence for unintended benziso­thia­zolinone 1-oxide formation from benzo­thia­zinones through oxidation
Source: Acta Crystallogr C Struct Chem. 2020 Aug 21;76(Pt 9):907–13. doi: 10.1107/S2053229620010931 (PMC7474187; doi:10.1107/S2053229620010931)
Supplement: Supplementary file 6 [file c-76-00907-sup6.pdf]

STRUCTURAL
CHEMISTRY

Volume 76 (2020)

Supporting information for article:

Crystallographic evidence for unintended benzisothiazolinone 1-oxide formation from benzothiazinones through oxidation

Tamira Eckhardt, Richard Goddard, Christoph Lehmann, Adrian Richter, Henok Asfaw Sahile, Rui Liu, Rohit Tiwari, Allen G. Oliver, Marvin J. Miller, Rüdiger W. Seidel and Peter Imming

Supporting information for

Crystallographic evidence for unintended benzisothiazolinone 1-oxide formation from BTZs through oxidation

Tamira Eckhardt, Richard Goddard, Christoph Lehmann, Adrian Richter, Henok Asfaw Sahile, Rui Liu, Rohit Tiwari, Allen G. Oliver, Marvin J. Miller, Rüdiger W. Seidel and Peter Imming

Description of the deliberate synthesis of 2

Compound **2** was prepared deliberately according to the sequence depicted in Figure S1. The synthesis of 2-chloro-5-trifluoromethyl-3-nitrobenzamide is described elsewhere [Cooper, M., Zuegg, J., Becker, B. & Karoli, T. (2013). *Eur. Pat. Appl.* EP 2570413 A1]. Reagents were purchased and used as received. Solvents were of reagent grade and distilled before use.

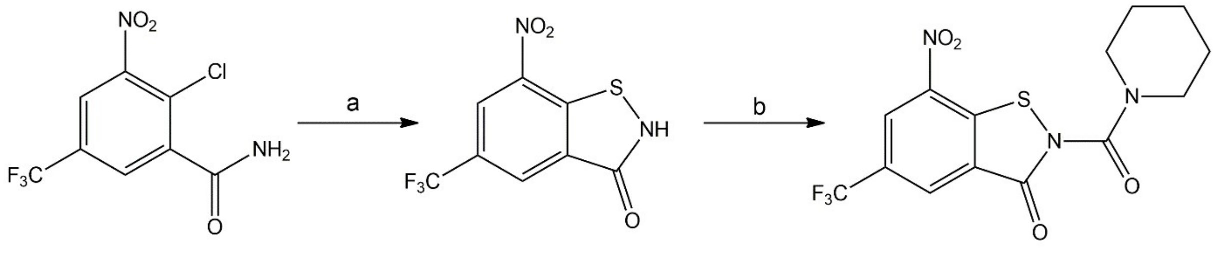

2

Figure S1. Deliberate synthesis of **2**; a: elemental sulfur; CuI, K_2CO_3, 1,10-phenanthroline, DMF, b: piperidine, triphosgene, trimethylamine, DCM.

7-Nitro-5-(trifluormethyl)benzisothiazolinone: copper(I) iodide (95 mg, 0.5 mmol) and 1,10-phenanthroline (90 mg, 0.5 mmol) in 5 mL of DMF were stirred in an argon atmosphere for 15 min. 2-Chloro-5-trifluoromethyl-3-nitrobenzamide (537 mg, 1.1 mmol), sulfur (80 mg, 1.3 mmol) and anhydrous K_2CO_3 (360 mg, 1.6 mmol) were added. After stirring for another 15 min, the mixture was refluxed overnight. For workup, 20 mL of brine were added. After stirring for 3 hours, the aqueous phase was extracted three times with 20 mL of ethyl acetate. The combined organic phases were dried over Na_2SO_4. Final purification was carried out by flash chromatography on silica gel, eluting with a gradient of chloroform-methanol 100 to

10 %. Yield, 101 mg (35%). R_f = 0.25 (uncorrected, silica gel, chloroform-methanol 9:1). MS(ESI$^-$): *m/z* 263.28 [M]$^-$. ^{1}H NMR ($CDCl_3$) δ 8.81 (s, 1H), 8.67 (s, 1H), 4.29 (broad s, 1H). ^{13}C NMR (100 MHz, $CDCl_3$) δ 164.7, 145.8, 141.2, 128.1 (q, $^2J_{C,F}$ = 34.3 Hz), 127.7, 123.0 (q, $^1J_{C,F}$ = 271.8 Hz), 122.9 ppm.

7-Nitro-2-(piperidine-1-carbonyl)-5-(trifluoromethyl)benzisothiazolinone (2): piperidine (0.016 g, 0.19 mmol) in 5 mL of dichloromethane (DCM) was added to a solution of triphosgene (0.056 g, 0.19 mmol) in 5 mL of DCM. Triethylamine (0.057 ml) in 0.5 mL of DCM was added dropwise. The mixture was stirred for 30 min, the solvent evaporated under reduced pressure, the residue redissolved in 10 mL of DCM and the solution added to a solution of 7-nitro-5-(trifluoromethyl)benzisothiazolone (0.05 g, 0.19 mmol) in 0.5 mL of tetrahydrofuran. After stirring the mixture for 16 hours, the same amount of the piperidine-triphosgene reagent was prepared and added. After removal of the solvents under reduced pressure, the residue was subjected to column chromatography, eluting with heptane 100 % to heptane/ethyl acetate 1:1. Compound **2** was isolated as a yellow solid (9 mg, 13%). ^{1}H NMR and HRMS data agreed with those for **2** obtained by treatment of BTZ **1** with 3-chloroperbenzoic acid.

Description of the anti-mycobacterial assays

Mycobacterium tuberculosis **H37Rv (harbouring RFP-expressing pTEC27 plasmid):** The plasmid confers resistance to hygromycin. The mycobacterial strains was routinely grown in 7H9 broth (Difco Middlebrook) supplemented with 10 % (v/v) OADC (5 % bovine albumin fraction, 2 % dextrose, 0.004 % catalase, 0.05 % oleic acid and 0.8 % sodium chloride solution) and 0.05 % (v/v) polysorbate 80 at 37 °C in standing cultures. Hygromycin B was added to the medium at a final concentration of 50 µg/mL.

MIC_{90} was determined by the broth microdilution method using a flat-bottom 96-well Corning Costar plates. In the first well in each row, two times the desired highest concentration (200 µg/mL) of each compound was added in 7H9 medium supplemented with 10 % OADS, and 0.05 % polysorbate 80 and hygromycin (50 µg/mL). Each well was then diluted twofold in a ten-point serial dilution. Subsequently, 100 µL of the bacterial inoculum were added to each well to give a final volume of 200 µL. The concentration of the inoculum of 5×10^5 cells/mL (OD_{600}, 0.1 = 0.33×10^8 CFU/mL) was prepared from a starting inoculum

that was diluted from a preculture at the mid-log phase (OD_{600}, 0.3 to 0.7). In each plate a negative control (1 % DMSO) and a positive control (100 µg/mL gentamicin) were included. The plates were sealed with parafilm, placed in a container with moist tissue and incubated for six days at 37 °C. After incubation the fluorescence intensity of each well was measured (Synergy H4 plate reader (BioTek), excitation λ = 530 nm, emission λ = 590 nm) and the MIC_{90} was calculated as the concentration of the compound that caused more than 90 % growth reduction. The determination was done in duplicate.

***Mycobacterium aurum* (DSMZ 43999):** MICs were determined by the broth microdilution method. 96-well flat bottom tissue culture plates (Sarstedt, 83.3924.500) were used. In the second well of each row two times the desired highest concentration of each compound was added in 7H9 medium supplemented with 10 % OADC and 0.5 % glycerol. Each compound was diluted twofold in a ten-point serial dilution. The concentration of the starting inoculum was 5×10^5 cells/mL. The starting inoculum was diluted from a preculture at the mid-log phase (OD_{600} 0.3 to 0.7) and a OD_{600} of 0.1 was correlated to 1×10^8 CFU/mL by a colony counting experiment. The plates were sealed with parafilm, put in a container with moist tissue and incubated for four days at 37 °C with shaking. Each plate had eight negative controls (1 % DMSO) and eight positive controls (100 µM isoniazid). After incubation the plates were monitored by OD measurement at 590 nm (BMG Novostar). The assay was performed in duplicate.

Every assay plate contained eight wells with DMSO (1 %) as negative control, which corresponds to 100 % bacterial growth and eight wells with isoniazid (100 µM) as positive control in which 100 % inhibition of bacterial growth was reached. Controls were used to monitor the assay quality through determination of the Z’ score. The Z’ factor was determined using the formula:

$$Z' = 1 - \frac{3(\mathrm{SD}_{\mathrm{isoniazid}} + \mathrm{SD}_{\mathrm{DMSO}})}{(\mathrm{M}_{\mathrm{isoniazid}} - \mathrm{M}_{\mathrm{DMSO}})}$$

(SD = standard deviation, M = mean)

The percentage of growth inhibition was calculated as follows:

$$\text{growth inhibition (\%)} = (-100)\,x\,\frac{(\mathrm{Signal}_{\mathrm{sample}} - \mathrm{Signal}_{\mathrm{DMSO}})}{(\mathrm{Signal}_{\mathrm{DMSO}} - \mathrm{Signal}_{\mathrm{sample}})}$$
